# Editorial: Methods and applications in computational physiology and medicine

**DOI:** 10.3389/fphys.2022.1092227

**Published:** 2022-11-24

**Authors:** A. H. Khandoker, P. Castiglioni, J. L. Greenstein, J. Zhao, F. S. Schlindwein, M. Elgendi, R. L. Winslow, Z. R. Struzik

**Affiliations:** ^1^ Department of Biomedical Engineering, Khalifa University, Abu Dhabi, United Arab Emirates; ^2^ Fondazione Don Carlo Gnocchi Onlus (IRCCS), Milan, Italy; ^3^ Department of Biomedical Engineering and Institute for Computational Medicine, Johns Hopkins University, Baltimore, MD, United States; ^4^ Auckland Bioengineering Institute, The University of Auckland, Auckland, New Zealand; ^5^ School of Engineering and NIHR Biomedical Research Unit, University of Leicester, Leicester, United Kingdom; ^6^ Biomedical and Mobile Health Technology Lab, ETH Zurich, Zurich, Switzerland; ^7^ Northeastern University, Boston, MA, United States; ^8^ Graduate School of Education, The University of Tokyo, Tokyo, Japan

**Keywords:** in silico, physiology, computational, medicine, modelling, multidisciplinary, methods

Functional complexity of bodily organs and their physiological functions, in particular in pathological conditions, continues to challenge fundamental research and medical practice. Recent decades established the role of computational methods for modelling all levels of physiological complexity starting from heuristic approaches to the rapidly gaining popularity data based modelling.

Understanding the complex physiological functions at different levels requires collaborative efforts from scientists with expertise in a wide range of disciplines—hence, contributions based on multidisciplinary approaches were encouraged for our edited volume. As a result, this special issue includes innovative methods and applications from organ to the sub-cellular level, biology to mathematics and physics, and computational models to new devices or systems.

Given the popularity of research on virtual environment platforms, the review article (Souza and Naves) summarised the state-of-the-art techniques for attention detection in virtual environments using EEG signal-based feature extraction methods and their interpretations. Of particular interest to the editors of this specialty were noninvasive and non-intrusive applications, approaches, and methods for better exploring and understanding complex physiological functions and their computational framework. This review could benefit future prospective research studies planning to conduct EEG-based research experiments in Virtual Reality.

Assessing the validity of a clinical model may be particularly difficult when it is not possible to determine causal links with traditional experimental methods. This is the problem that Pinna and Maestri had to face to verify the hypothesis that during Cheyne-Stokes respiration in heart failure patients with central apneas, the increases in ventilation induced by arousal from sleep may sustain or even exacerbate the ventilatory oscillation.

Models of computational fluid dynamics are of paramount importance for understanding cardiovascular hemodynamics. In their paper, Qiao et al. present a computational model of thrombosis and apply it to simulate the process of thrombus formation in the U.S. Food and Drug Administration (FDA) benchmark nozzle. The similarity between experimental results and computational prediction confirms the accuracy of their model, which may help to understand better how hemolysis processes occur at different flow regimes and the effects of turbulence on thrombosis.

Kim et al. use *in silico* modelling to address a complex pathophysiological question of the possible underpinning of potentially fatal changes in the electrophysiology of vital cardiac signals. To identify possible suspects in terms of the contributing individual ionic currents, Kim et al. investigated the impact of reduced hybrid/complex N-glycosylation through the lens of electrophysiology and *in silico* modelling—aiming at fitting the known models to their experimental results. The authors developed a metaheuristics optimisation method based on a genetic algorithm (G.A.) to calibrate the computer models—they claim that their approach facilitates the prediction of individual component current characteristics at different voltages, which are not directly observed in the *in vitro* experiments.

Better understanding human cardiac cellular structure in healthy and disease conditions will improve our modelling approach and its reliability for predicting potential pathology. Giardini et al. developed an integrative approach by combining tissue clearing, staining, and microscopy techniques to detect and characterise sarcomeres in human myocardial tissue and test in both healthy and pathological remodelled human cardiac tissue.

The design and evaluation of medical devices benefit from the integration and application of various *in silico* approaches before any *in vivo* experiments are undertaken. In the case of microcirculatory support (MCS) devices, Ozturk et al. design and apply a multi-domain computational framework to develop and characterise a pulsatile-flow MCS device for heart failure with preserved ejection fraction. The *in silico* results demonstrated that the pulsatile pump design restores left heart pressures and wall stresses to physiological levels in this model of heart failure, which could potentially alleviate associated remodelling processes and symptoms. In addition, the results represent a promising step in the direction of improved device performance and patient outcomes as they demonstrate that pulsatile-flow support yields more physiological arterial hemodynamics when compared with current continuous-flow MCS protocols in this heart failure phenotype.

The application of machine learning and artificial intelligence in clinical diagnostic tasks is an area of great interest and rapid growth in making personalised medicine attainable. With the successful development of potent neural network-based algorithms for image classification and segmentation, pathological studies where histological samples must be quantitatively assessed naturally benefit from these advances. Thorsted et al. hypothesise that artificial intelligence can support high throughput analyses of histological sections of excised human abdominal aortic aneurysms (AAA) and develop a pipeline to achieve this.

New insights in computational physiology and medicine are often derived from applying one or a combination of standard techniques and methods to a currently unaddressed question. Less often, however, are we asked to think about the problem at hand by stepping back from our go-to toolbox of methods and looking at it through another lens. Hussan et al. do this by modelling across the multiple spatial and temporal scales of biological hierarchy. By drawing on the framework of statistical physics, the authors address the complexity question by recognising maximum entropy to be a principle that guides multicellular biophysics. The prototype model reveals physiologically relevant emergent behaviour that would not be expected in traditional models of the same scale.

In summary, this edited volume proves that methods are vital for innovation in the field of Computational Physiology and Medicine. It also highlighted the transitions the field is undergoing in respect to the methodological approaches. While increasing sophistication drives specialised expertise, multidisciplinary approaches become indispensable for “joining the dots.” Where heuristic, phenomenology driven insight becomes hampered and models are too difficult to build or are simply unknown, data driven modelling is increasingly used. Isolated phenomena become more frequently cross-coupled or embedded in the system to account for the emergent complexity. These advances in methodological approaches provide great opportunities to increase the clinical availability of practical health applications and enhance the accuracy of clinical decision-making. We are looking forward to the follow up in these exciting developments.

